# Ubiqutination via K27 and K29 chains signals aggregation and neuronal protection of LRRK2 by WSB1

**DOI:** 10.1038/ncomms11792

**Published:** 2016-06-07

**Authors:** Frederick C. Nucifora, Leslie G. Nucifora, Chee-Hoe Ng, Nicolas Arbez, Yajuan Guo, Elaine Roby, Vered Shani, Simone Engelender, Dong Wei, Xiao-Fang Wang, Tianxia Li, Darren J. Moore, Olga Pletnikova, Juan C. Troncoso, Akira Sawa, Ted M. Dawson, Wanli Smith, Kah-Leong Lim, Christopher A. Ross

**Affiliations:** 1 Department of Psychiatry and Behavioral Sciences, Johns Hopkins University School of Medicine, Baltimore, Maryland 21287, USA; 2 Danone Nutricia Research, 30 Biopolis Street, Matrix Building, #05-01B, Singapore 138671, Singapore; 3 Department of Molecular Pharmacology, Rappaport Institute of Medical Research, Technion-Israel Institute of Technology, Haifa 31096, Israel; 4 Department of Pharmaceutical Sciences, University of Maryland School of Pharmacy, Baltimore, Maryland 21201, USA; 5 Center for Neurodegenerative Science, Van Andel Research Institute, Grand Rapids, Michigan 49503, USA; 6 Division of Neuropathology, Department of Pathology, Johns Hopkins University School of Medicine, Baltimore, Maryland 21201, USA; 7 Department of Neurology, Johns Hopkins University School of Medicine, Baltimore, Maryland 21201, USA; 8 Solomon H. Snyder Department of Neuroscience, Johns Hopkins University School of Medicine, Baltimore, Maryland 21201, USA; 9 Neuroregeneration and Stem Cell Programs, Institute for Cell Engineering, Johns Hopkins University School of Medicine, Baltimore, Maryland 21201, USA; 10 Department of Pharmacology and Molecular Sciences, Johns Hopkins University School of Medicine, Baltimore, Maryland 21201, USA; 11 Adrienne Helis Malvin Medical Research Foundation, New Orleans, Louisiana 70130-2685, USA; 12 Neuroscience and Behavioral Disorders Program, Duke-National University of Singapore Graduate Medical School, Singapore 169857, Singapore; 13 Department of Physiology, National University of Singapore, Singapore 117543, Singapore

## Abstract

A common genetic form of Parkinson's disease (PD) is caused by mutations in LRRK2. We identify WSB1 as a LRRK2 interacting protein. WSB1 ubiquitinates LRRK2 through K27 and K29 linkage chains, leading to LRRK2 aggregation and neuronal protection in primary neurons and a *Drosophila* model of G2019S LRRK2. Knocking down endogenous WSB1 exacerbates mutant LRRK2 neuronal toxicity in neurons and the *Drosophila* model, indicating a role for endogenous WSB1 in modulating LRRK2 cell toxicity. WSB1 is in Lewy bodies in human PD post-mortem tissue. These data demonstrate a role for WSB1 in mutant LRRK2 pathogenesis, and suggest involvement in Lewy body pathology in sporadic PD. Our data indicate a role in PD for ubiquitin K27 and K29 linkages, and suggest that ubiquitination may be a signal for aggregation and neuronal protection in PD, which may be relevant for other neurodegenerative disorders. Finally, our study identifies a novel therapeutic target for PD.

## Abstract (web summary)

Mutations in LRRK2 are linked to Parkinson's Disease. Here, the authors identify WSB1 as a LRRK2 interacting protein and find that it promotes LRRK2 aggregation in primary neurons and drosophila models via ubiquitin K27 and K29 linkages.

## Graphical abstract

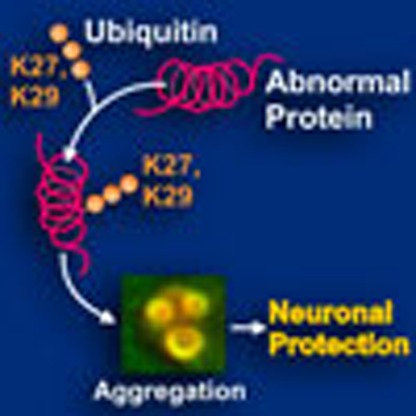

Parkinson's disease (PD) is a progressive neurodegenerative disorder characterized by tremor, rigidity and bradykinesia, with no known disease-modifying treatment. Neuropathology is characterized by selective neuronal cell death^1^,^2^ and intracellular cytoplasmic aggregates termed Lewy bodies^3^. Lewy bodies and related Lewy neurites contain alpha-synuclein and ubiquitin, and may represent a protective response by neurons to toxic proteins^4^.

The ubiquitin proteasome system (UPS) is responsible for protein degradation within the cell^5^. Proteins fated for degradation are tagged with ubiquitin through the formation of an iso-peptide bond between the ɛ-amino group of a lysine residue of the substrate and the C-terminal carboxylate of ubiquitin. This ligation reaction is a process requiring a repeated series of actions involving ubiquitin-activating (E1), -conjugating (E2) and -ligating (E3) enzymes, polyubiquitinating a protein^6^. Once a protein is ubiqutinated it is targeted for degradation or one of several possible cellular processes.

Ubiquitin is a 76 amino acid protein that contains 7 lysines. The cellular process activated is thought to be determined by which ubiquitin lysine is utilized to congregate ubiquitin to the target protein^7^. The most studied process to date is the linkage through K48, which leads to protein degradation through the proteasome^8^. Data suggest that K63 may be involved in DNA repair^7^,^9^, endocytosis^7^,^9^ and NFκB signalling^9^,^10^. Much less is known about the functioning of the other lysine linkages.

Dysfunction of the UPS has been implicated in both genetic and sporadic forms of PD^11^,^12^. In brains from PD patients, ubiquitinated proteins and components of the UPS appear in Lewy bodies^11^,^12^. Proteasomal inhibition itself may cause the formation of protein inclusions and lead to degeneration of neurons in the substantia nigra in rats^13^,^14^. Parkin is an E3 ligase^15^,^16^,^17^ and has been shown to function as part of an E3 ligase complex with the PTEN-induced kinase (PINK1) and DJ-1 (ref. 18).

A major genetic form of PD is caused by mutations in the leucine-rich repeat kinase 2 protein (LRRK2)^19^,^20^. Several different point mutations segregate with PD^21^,^22^,^23^, and the point mutations are present in almost all of the identified domains^24^,^25^,^26^,^27^,^28^. The LRRK2 G2019S mutation is the most common and accounts for ∼1% of sporadic PD and up to 25% of familial PD in certain populations^29^,^30^. The clinical phenotype and pathological changes associated with LRRK2 mutations are generally similar to sporadic PD^19^,^20^. The distribution of mutations in several different domains, and the lack of deletions or truncations, along with dominant inheritance, are consistent with a gain of function mechanism. This gain of function could involve alterations of a normal function, such as protein interaction or protein kinase activity. There could also be a gain of a novel adverse function.

LRRK2 is a very large protein of 2,527 amino acids, containing several putative domains^31^. LRRK2 includes the Roc (Ras in complex proteins), belonging to the Ras/GTPase family, and a COR domain (C-terminal of Roc) together making a GTP-binding regulatory domain. It also has a kinase effector domain (MAPKKK), and two consensus protein interacting domains, a leucine-rich repeat (LRR), consisting of 12 repetitions of a 22–28 amino acid motif, and a WD40 domain^32^.

Understanding of LRRK2 pathogenesis would be greatly enhanced by improved understanding of LRRK2 regulation and binding proteins^33^. We have previously shown that the carboxy terminus of Hsp70-interacting protein (CHIP) interacts with and ubiquitinates LRRK2 and promotes proteasomal degradation of LRRK2 (ref. 34). To identify additional interactors, we performed a yeast two-hybrid screen, and identified WD repeat and SOCS box-containing protein 1 (WSB1). WSB1 is part of an E3 ubiquitin ligase complex for the thyroid hormone activating type-2 iodothyronine deiodinase^35^. WSB1 is also an E3 ligase that can modulate apoptosis via Homeodomain-interacting protein kinase 2 (ref. 36).

In the present study, we show that WSB1 is a novel interacting protein of LRRK2. WSB1 ubiquitinates LRRK2 through ubiquitin chains, K27 and K29, leading to LRRK2 aggregation and neuronal protection in both primary neurons and a *Drosophila* model of LRRK2 PD. WSB1 is found in Lewy bodies in human PD post-mortem tissue. These data indicate a role for WSB1 in modulation of mutant LRRK2 pathogenesis, and an involvement in Lewy body pathology in sporadic PD. The data also suggest that K27 and K29 ubiquitin linkages constitute a signal for protein aggregation. Overall, our data suggest a cell survival pathway that may be involved not just in PD, but also other neurodegenerative disorders. Furthermore, our data identify a possible therapeutic target.

## Results

### LRRK2 interacts with WSB1

**Figure 1:**
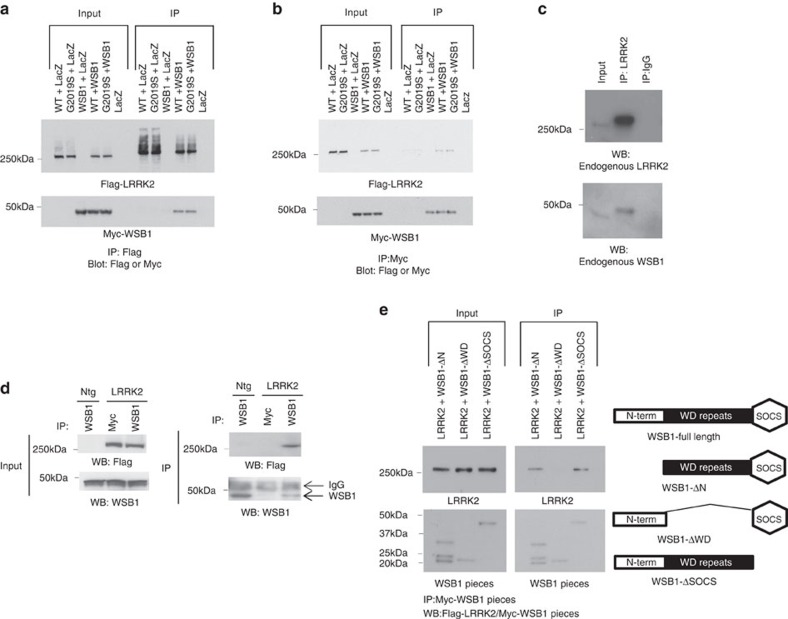
LRRK2 interacts with WSB1 in cells and *in vivo*. (**a** and **b**) Co-Immunoprecipitation of WT LRRK2 or G2019S LRRK2 and WSB1 transfected into HEK293 cells. (**c**) Co-Immunoprecipitation of endogenous LRRK2 and endogenous WSB1 in NIH 3T3 mouse embryonic fibroblast cells. (**d**) Co-immumopreciptiation of LRRK2 and WSB1 from LRRK2 transgenic mice. (**e**) Deletion mutants lacking the consensus sites of WSB1 were generated and co-immunoprecipitation of LRRK2 with the individual deletion mutations of WSB1 were performed. These data show that WSB1 interacts with LRRK2 and that the interaction occurs through the WD domain of WSB1.

To confirm that LRRK2 interacts with WSB1, we co-transfected full-length LRRK2 with full-length WSB1 in HEK 293 cells, and showed that WSB1 and LRRK2 specifically co-immunoprecipitated (Fig. 1a,b). We also demonstrate that endogenous LRRK2 and endogenous WSB1 interact in NIH 3T3 mouse embryonic fibroblast cells (Fig. 1c). Immunoprecipitation for WSB1 using a transgenic mouse model expressing full-length Flag-tagged LRRK2 (manuscript in preparation) demonstrated an *in vivo* interaction (Fig. 1d) between these proteins. Supplementary Fig. 1A shows the generation of a peptide antibody specific for WSB1. These data confirm an interaction between LRRK2 and WSB1.

To provide evidence of a direct interaction and better define the interaction domain of WSB1 with LRRK2, we made mutants of WSB1 lacking the consensus domains: the N terminus, the WD repeat domain or the SOCS domain, and determine which region was no longer able to bind. When the WD repeat domain was removed, WSB1 no longer interacted with LRRK2, suggesting that the interaction occurs at the WD domain of WSB1 (Fig. 1e).

### WSB1 ubiquitinates LRRK2

**Figure 2:**
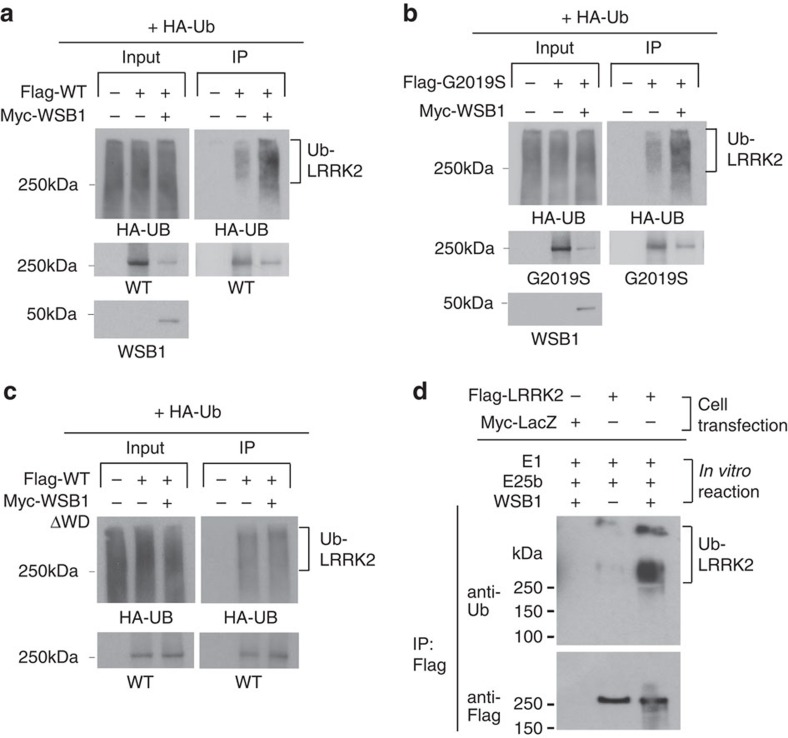
WSB1 ubiquitinates LRRK2. *In vivo* ubiquitination assay. (**a**,**b**) HA-tagged ubiquitin alone, or co-transfected with LRRK2, with or without WSB1. LRRK2 was immunoprecipitated and western blots were performed to identify the amount of HA-ubiquitin detected. These experiments demonstrate that WSB1 ubiquitinates LRRK2. (**c**) Ubiquitin assay performed above using the WSB1-ΔWD construct that does not interact with LRRK2. This demonstrates that WSB1 cannot increase ubiquitination when it does not interact with LRRK2. (**d**) *In vitro* ubiquitin assay demonstrating that WSB1 is an E3 ligase for LRRK2.

Since WSB1 has been reported to be an E3 ligase, we conducted a cellular ubiquitination assay, (Fig. 2a,b) and demonstrated that LRRK2 was robustly labelled with ubiquitin when co-transfected with WSB1. By contrast, when we performed the same assay but used the WSB1-ΔWD construct that does not interact with LRRK2 there was no increase in ubiquitination compared with LRRK2 alone (Fig. 2c). An *in vitro* ubiquitination assay confirmed that LRRK2 is ubiquitinated in the presence of recombinant E1, E2 and WSB1 (Fig. 2d), indicating that WSB1 functions directly as an E3 ligase for LRRK2.

### WSB1 ubiquitinates LRRK2 via atypical chains K27 and K29

**Figure 3:**
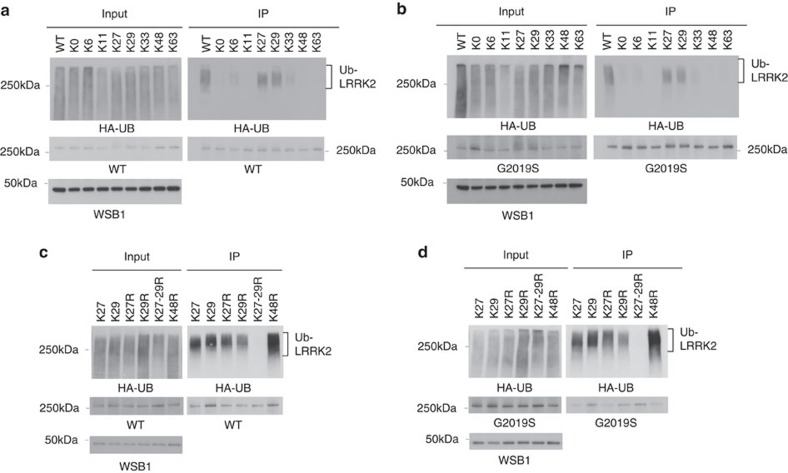
WSB1 ubiquitinates LRRK2 through K27 and K29 linkages. (**a**,**b**) HA-tagged ubiquitin mutants with only the indicated lysine residue available for chain formation were used and an *in vivo* ubiquitin assay was performed. (**c**,**d**) On the basis of the results from **a** and **b**, we mutated the lysine at K27, K29 or K27 and K29 (K27R, K29R and K27–29R) to arginine and performed the *in vivo* ubiquitin assay. These experiments demonstrate that WSB1 ubiquitinates LRRK2 primarily through K27 and K29 lysine linkages.

We then sought to determine which ubiquitin linkage was occurring when WSB1 ubiquitinated LRRK2 (Fig. 3a,b). We conducted a cellular ubiquitination assay, by cotransfecting LRRK2 and WSB1 with individual ubiquitin constructs that could only form ubiquitin linkages at a single lysine (for example, K48 indicates every lysine except K48 is changed to argenine). A construct was used that represented each of the seven individual lysines in ubiquitin and K0 had no lysines available. Figure 3a,b demonstrate that WSB1 ubiquitinates both wild-type (WT) and G2019S LRRK2 primarily through chains K27 and K29.

To further establish these significant linkages, we mutated the lysines at K27 alone, K29 alone or both K27 and K29 to arginines (so that every lysine except K27 and K29 was now functional) and performed the assay described above (Fig. 3c,d). We see that when K27 and K29 are both mutated to arginine (K27-29R) WSB1 no longer ubiquitinates WT or G2019S LRRK2. Interestingly, K48 does not show any evidence of ubiquitination, and K48R does not decrease ubiquitination, suggesting that WSB1 does not target LRRK2 to the proteasome for degradation. Therefore, these data demonstrate that K27 and K29 are the predominant linkages.

### WSB1 alters the levels of soluble LRRK2

**Figure 4:**
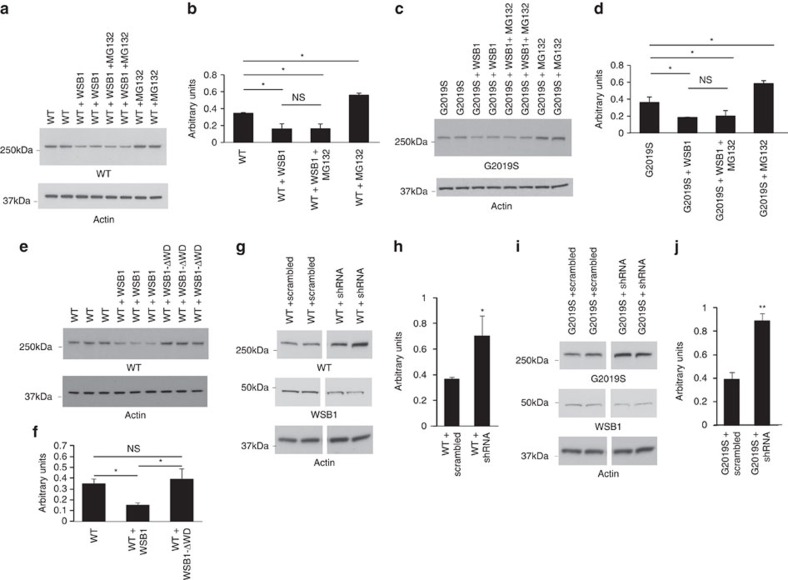
WSB1 reduces the levels of soluble LRRK2. (**a**,**c**) N2a neuroblastoma cells were transfected with LRRK2 alone or with WSB1 with and without the proteasome inhibitor MG132. Cell lysates were run on an SDS-PAGE gel and Western blotted for LRRK2. (**b**,**d**) Quantification of **a** and **c**, respectively. These data demonstrate that WSB1 decreases soluble LRRK2 but not through proteasomal degradation. (**e**) N2a neuroblastoma cells were transfected with LRRK2 alone or with WSB1 or WSB1-ΔWD. (**f**) Quantification of **e**. These data show that WSB1-ΔWD, which does not interact with LRRK2 was unable to reduce the levels of LRRK2. (**g**,**i**) N2a cells were transfected with LRRK2 and shRNA to WSB1 or scrambled shRNA. (**h**,**j**) Quantification of **g** and **i**. These data demonstrate that knocking down WSB1 increases LRRK2 soluble levels. All experiments were performed on at least three independent experiments where data are the mean±s.d. *P<0.05, **P<0.001 using a one-way analysis of variance test.

Soluble LRRK2 protein levels were significantly decreased when WSB1 was co-expressed with either WT or mutant LRRK2, but unchanged when LRRK2 was co-expressed with beta-galactosidase in N2A cells (Fig. 4a–d) or in HEK293 cells (Supplementary Fig. 1b–e). There was no significant increase in LRRK2 protein levels in this experiment in the presence of MG132, again suggesting that WSB1 is not decreasing LRRK2 levels through proteasomal degradation in N2a cells (Fig. 4a–d) or in HEK293 cells (Supplementary Fig. 1b–e). MG132 does significantly increase the levels of LRRK2 when not in the presence of WSB1 further suggesting that WSB1 is not decreasing LRRK2 levels through proteasomal degradation (Fig. 4a–d) and confirming MG132 is capable of blocking degradation of LRRK2 alone. Figure 4e,f shows that co-expression of LRRK2 and WSB1-ΔWD, the construct that does not interact with LRRK2, in N2a cells does not decrease the expression of LRRK2. We also show that when WSB1 is knocked down by short hairpin RNA (shRNA) in N2a cells, LRRK2 levels significantly increase, further suggesting that WSB1 modulates LRRK2 (Fig. 4g–j).

### WSB1 leads to LRRK2 aggregation

Since we observed a decrease in protein expression in the soluble LRRK2 levels, not dependent on proteasomal degradation, we hypothesized that WSB1 may cause LRRK2 to become insoluble. We performed a sarkosyl detergent fractionation experiment in N2a cells, using a series of detergents and gradients to fractionate proteins into soluble and insoluble fractions.

**Figure 5:**
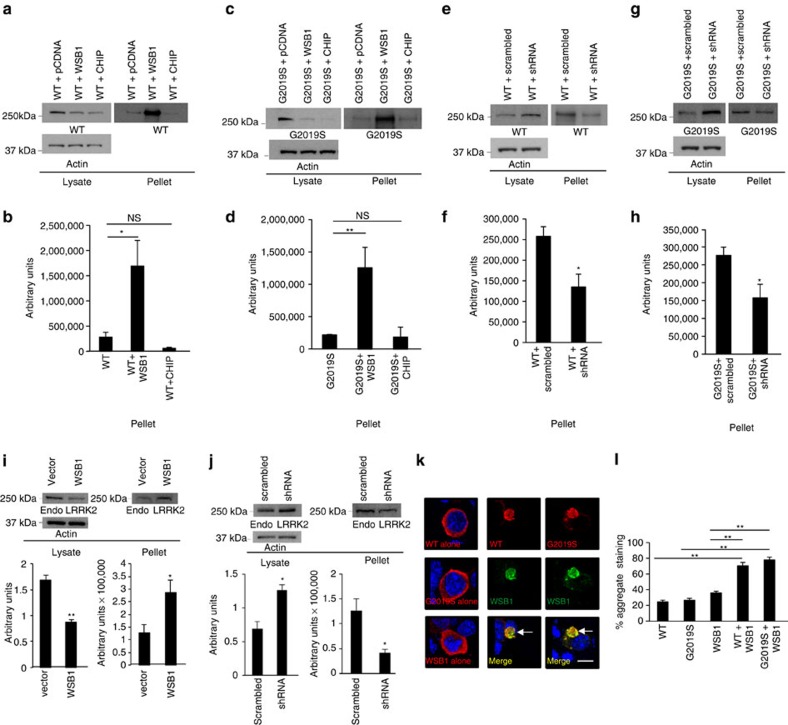
WSB1 leads to LRRK2 aggregation. (**a**,**c**) Sarkosyl detergent fractionation from N2A cells co-transfected with LRRK2 plus pcDNA, WSB1 or CHIP. (**b**,**d**) Quantification of experiments from A and C. WSB1 significantly increases LRRK2 insolubility demonstrated by WSB1 significantly increasing the amount of LRRK2 in the insoluble fraction. (**e**,**g**) Sarkosyl detergent fractionation from N2A cells cotransfecting WT or G2019S LRRK2 and shRNA to WSB1 or scrambled shRNA. (**f**,**h**) Quantification of experiments from **e** and **g**. Knocking down WSB1 decreases LRRK2 in the insoluble fraction and increases LRRK2 in the soluble fraction. (**i**) Sarkosyl detergent fractionation in NIH 3T3 cells transfected with WSB1. WSB1 significantly decreases soluble endogenous LRRK2 (**i**, left) and significantly increases insoluble endogenous LRRK2 (**i**, right). (**k**) Sarkosyl detergent fractionation in NIH 3T3 cells transfected with shRNA to WSB1. Knocking down endogenous WSB1 increased soluble endogenous LRRK2 (**j**, left) and decreased insoluble endogenous LRRK2 (**j**, right). These data demonstrate that WSB1 can modulate the solubility and insolubility of endogenous LRRK2. (**k**) Immunocytochemistry of transfected LRRK2 alone, WSB1 alone, or with LRRK2 plus WSB1 in N2A cells. Blue staining in images represents 4,6-diamidino-2-phenylindole (DAPI) stain. Scale bar, 10 μm. (**l**) Quantification of aggregate like staining from immunocytochemistry in **k**. WSB1 significantly increases LRRK2 aggregation. All experiments were performed on at least three independent experiments where data are the mean±s.d. *P<0.05, **P<0.001 using a one-way analysis of variance test.

Separation of homogenate into soluble and insoluble fractions shows that both WSB1 and CHIP decrease LRRK2 expression in the soluble fraction. However, unlike CHIP, which has been shown to decrease LRRK2 expression through proteasomal degradation, WSB1 markedly increases the amount of WT and G2019S LRRK2 in the insoluble fraction (Fig. 5a–d). This suggests that WSB1 acts via a different mechanism from CHIP, and does not lead to protein degradation, but to LRRK2 aggregation.

Performing the same assay as described above but using the WSB1-ΔWD construct showed no change in soluble or insoluble levels of LRRK2 as would be expected since this construct does not interact with LRRK2 (Supplementary Fig. 1F,G)

We then performed the sarkosyl fractionation experiment using shRNA to WSB1 in N2a cells. We show that when WSB1 is knocked down, LRRK2 is significantly decreased in the insoluble fraction, and is increased in the soluble fraction (Fig. 5e–h). By contrast scrambled shRNA has no effect. This further suggests that WSB1 causes LRRK2 aggregation.

To determine the effects of WSB1 on endogenous LRRK2 solubility and insolubility we performed sarkosyl fractionation in NIH 3T3 cells. We demonstrate that WSB1 significantly decreases soluble endogenous LRRK2 (Fig. 5i left) and significantly increases insoluble endogenous LRRK2 (Fig. 5i right). We also performed this experiment using shRNA to WSB1 in NIH 3T3 cells and show that knocking down endogenous WSB1 increased soluble endogenous LRRK2 (Fig. 5j left) and decreased insoluble endogenous LRRK2 (Fig. 5j right). These data demonstrate that WSB1 can modulate the solubility and insolubility of endogenous LRRK2.

In immunofluorescence experiments in N2A cells, most cells expressing LRRK2 or WSB1 transfected alone had diffuse cytoplasmic label for each protein alone. However, when LRRK2 and WSB1 were co-transfected, the proteins co-localized in aggregate structures (Fig. 5k,l). When WSB1 was knocked down in N2a cells, there is a significant decrease in the number of cells with aggregated structures (Supplementary Fig. 2A). These data provide cell biological confirmation that WSB1 leads to LRRK2 aggregation.

### WSB1 causes LRRK2 aggregation and rescues neuronal toxicity

**Figure 6:**
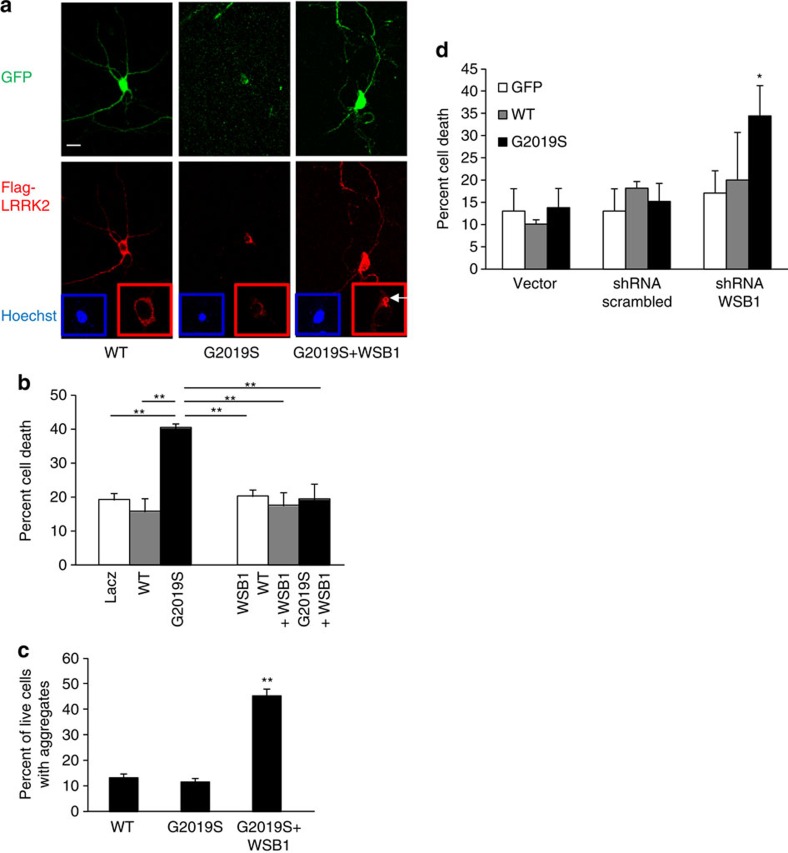
Gain and loss of function experiments indicate a role for WSB1 in modulation of LRRK2 toxicity in primary neurons. (**a**) Immunocytochemistry of primary cortical neurons transfected with GFP plus WT LRRK2, GFP plus G2019S LRRK2 or GFP plus G2019S LRRK2 plus WSB1. Inset blue shows Hoechst staining of nuclei and a condensed nucleus for G2019S but not for WT or G2019S plus WSB1. Red inset shows higher magnification and exposure optimum for cytoplasmic staining, demonstrating aggregate formation when WSB1 is co-transfected with G2019S (white arrow). Scale bar, 10 μm. (**b**) Quantification of cortical neuronal toxicity. Graph shows results of at least three independent experiments done in triplicate. Data are the mean±s.d. **P<0.001 using a one-way analysis of variance (ANOVA) test. (**c**) Quantification of aggregate structures in primary neurons. Graph shows results of two independent experiments done in quadruplicate. Data are the mean±s.d. **P<0.001 using a one-way ANOVA test. (**d**) Quantification of neuronal toxicity using shRNA to WSB1 or scrambled shRNA or vector control. Graph shows results of two independent experiments done in quadruplicate. Data are the mean±s.d. *P<0.05 using a one-way ANOVA test. These data demonstrate that WSB1 modulates LRRK2 aggregation and toxicity.

Since WSB1 ubiquitinated LRRK2, and led to protein aggregation, and because protein aggregation could be neuroprotective, we investigated the effect of WSB1 on mutant LRRK2 toxicity. Figure 6a shows immunolabel demonstrating abnormal neurite processes and nuclear condensation when mutant LRRK2 is expressed in neurons, indicating cellular toxicity. By contrast, there is rescue of this phenotype and formation of aggregate structures (white arrow), when WSB1 is co-expressed with mutant LRRK2. Quantification of toxicity is shown in Fig. 6b. Using a nuclear condensation assay (which we have shown to correlate with cell death^37^), we demonstrate that mutant but not WT LRRK2 caused significant neuronal cell toxicity at 48 h. Co-transfection with WSB1 markedly decreased mutant LRRK2 cell toxicity (Fig. 6b). We also show that when WSB1 is co-transfected with LRRK2 there is a significant increase in LRRK2 aggregation (Fig. 6c). These data are consistent with the idea that WSB1 rescues LRRK2 toxicity by causing LRRK2 to aggregate.

To determine the effect of endogenous WSB1 on LRRK2 toxicity, we used shRNA to knockdown endogenous WSB1. ShRNA to WSB1, but not scrambled shRNA or vector alone, significantly decreased WSB1 expression in primary neurons (Supplementary Fig. 2B). We then performed the nuclear condensation assay, and demonstrated that knocking down WSB1 with shRNA to WSB1 led to increased neuronal toxicity with mutant LRRK2, compared with mutant LRRK2 with scrambled shRNA or mutant LRRK2 plus vector (Fig. 6d). These experiments were performed at 24 h, a time point when mutant LRRK2 does not lead to neuronal toxicity. These data indicate that endogenous WSB1 modulates mutant LRRK2 toxicity.

### WSB1 rescues the abnormal phenotype in LRRK2 *Drosophila*

**Figure 7:**
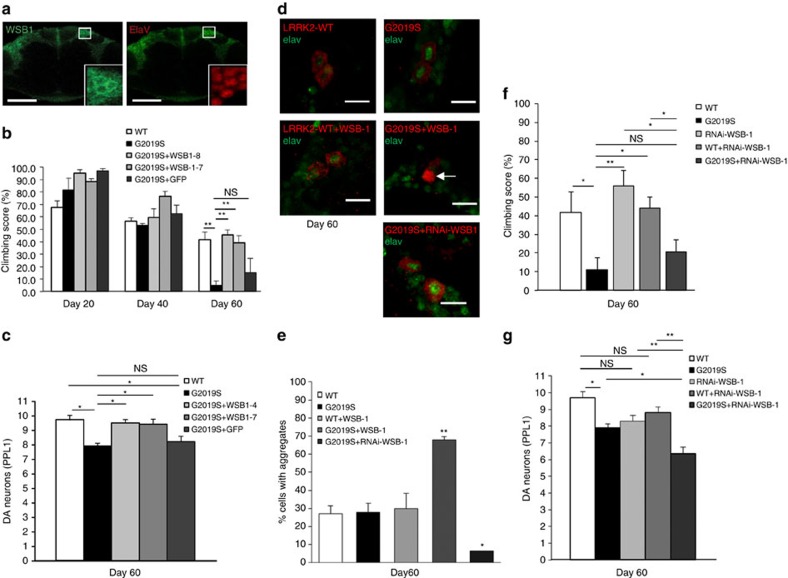
WSB1 modulates LRRK2 aggregation, neuronal toxicity, and climbing behaviour in LRRK2 *Drosophila* models. (**a**) Anti-WSB1 (green) and anti-elav (red) immunostaining of whole-mount adult brains derived from transgenic flies expressing wild type human WSB1 driven by the elav-promoter. *Inset*, Enlarged images from boxed region showing the localization of WSB1 and elav signals to the cytoplasm and nucleus, respectively. Scale bar, 50 μm. (**b**) Climbing score of Ddc-driven WT, G2019S, G2019S+WSB1 (lines 1–4 and 1–7) and G2019S+GFP-expressing flies at different age post-eclosion, as indicated. These data show that WSB1 rescues the climbing phenotype of G2019S *Drosophila*. (**c**) Quantification of TH+ neurons in the PPL1 cluster of WT, G2019S, G2019S+WSB1 (lines 1–4 and 1–7) and G2019S+GFP expressing flies at day 60. These data demonstrate that WSB1 rescues the neuronal toxicity in G2019S flies. (**d**) Immunocytochemistry of WT, G2019S, WT+WSB1, G2019S+WSB1 and G2019S+RNAi-WSB1-expressing *Drosophila* at day 60. Confocal images show aggregate formation in G2019S+WSB1 *Drosophila* (white arrow). Scale bar, 10 μm. (**e**) Quantification of **d**, indicating that there is a significant increase in aggregation in the G2019S+WSB1-expressing *Drosophila* and a significant decrease in aggregation in the G2019S+RNAi-WSB1-expressing *Drosophila*. These data together demonstrate that WSB1 modulates LRRK2 aggregation, toxicity and behavioral phenotype in an *in vivo* model. (**f**,**g**) WSB1 knockdown *Drosophila* mated to LRRK2 *Drosophila*. (**f**) Climbing score of WT, G2019S, RNAi-WSB1, WT+RNAi-WSB1, G2019S+RNAi-WSB1, expressing flies at day 60. (**g**) Quantification of TH+ neurons in the PPL1 cluster of WT-, G2019S-, RNAi-WSB1-, WT+RNAi-WSB1- and G2019S+RNAi-WSB1-, expressing flies at day 60. These data demonstrate that there is a significant increase in toxicity with G2019S+WSB1 RNAi compared to G2109S alone. In addition, there is a decrease in aggregation when WSB1 is knocked down (**d**,**e**). This further suggests that WSB1 regulates G2019S LRRK2 toxicity through protein aggregation. Data are the mean±s.d. *P<0.05, **P<0.001 using a one-way analysis of variance test.

To investigate the possible modulation of LRRK2 toxicity by WSB1 *in vivo*, we used an established *Drosophila* LRRK2 model^38^. We generated transgenic *Drosophila* over-expressing WSB1. When driven by the pan-neuronal *elav*-GAL4 driver, the expression of WSB1 in fly neurons is robust, and is localized predominantly to the cytoplasm (Fig. 7a). Whereas flies expressing G2019S alone exhibit significant climbing impairment at day 60 relative to control flies, the G2019S+WSB1 flies climb at levels comparable to control flies (Fig. 7b). In addition, WSB1 co-expression protected against the dopaminergic neuronal loss seen in the G2019S-expressing flies (Fig. 7c). We also see a significant increase in aggregates in the G2019S+WSB1 *Drosophila* (Fig. 7d,e). These data suggest that the rescue in *Drosophila* phenotype by WSB1 is related to aggregation formation. Flies expressing G2019S+GFP showed no significant difference in their ability to climb or in toxicity compared with flies expressing G2019S alone, suggesting that there are no titration effects from multiple upstream activation sequences (Fig. 7b,c). Supplementary Fig. 2C,D shows that the total levels of protein are not significantly different between *Drosophila* models used in these experiments. WSB1 flies alone showed no difference in ability to climb compared with control (Supplementary Fig. 2E).

We also generated WSB1 knockdown *Drosophila* and mated them to the LRRK2 *Drosophila*. Supplementary Fig. 2F,G shows that WSB1 RNA interference (RNAi) #1 decreases WSB1 expression by about 40% and RNAi #1 was used for all subsequent experiments. While we did not see a worsening of the climbing phenotype (Fig. 7f), Fig. 7g demonstrates that there is a significant increase in toxicity with G2019S+WSB1 RNAi compared with G2109S alone. Importantly, we do not see a significant increase in toxicity between WT alone and WT+WSB1 RNAi #1, suggesting that the increase in toxicity seen between G2019S and G2019S+WSB1 RNAi is due to WSB1's effect on mutant LRRK2. In addition, there is a decrease in aggregation when WSB1 is knocked down (Fig. 7d,e). This further suggests that WSB1 regulates G2019S LRRK2 toxicity through protein aggregation.

### WSB1 is present in Lewy bodies in human PD brain

**Figure 8:**
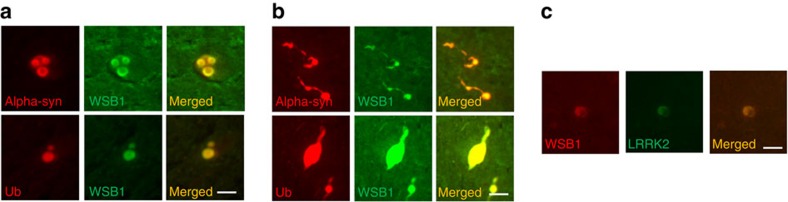
WSB1 is present in Lewy bodies in human PD post-mortem brain tissue. (**a**) PD post-mortem tissue from the substantia nigra, showing WSB1 co-localizes with the Lewy body markers, alpha-synuclein and ubiquitin in Lewy bodies. (**b**) PD post-mortem tissue from the substantia nigra, showing WSB1 co-localizes with alpha-synuclein and ubiquitin in Lewy neurites. (**c**) LRRK2 and WSB1 also co-localize in Lewy bodies. This suggests a role for WSB1 in sporadic PD, as well as LRRK2 PD. Scale bars, 10 μm.

Since WSB1 is present in aggregates in the cell models, we tested whether it would be in Lewy bodies in human PD brain. Notably, in human sporadic PD post-mortem substantia nigra, we found that WSB1 co-localizes with two markers (alpha-synuclein and ubiquitin) in Lewy bodies (Fig. 8a) and Lewy neurites (Fig. 8b). Furthermore, LRRK2 and WSB1 co-localized in Lewy bodies (Fig. 8c), further supporting that LRRK2 and WSB1 interact, and that WSB1 may play a role in PD pathology. 97% of the Lewy bodies identified by alpha-synuclein had WSB1 reactivity and 25% had LRRK2 (consistent with published reports)^39^. There were no Lewy bodies positive for LRRK2 but not WSB1. In addition, we stained Alzheimer's post-mortem brain tissue and did not detect any WSB1 reactivity in plaques, suggesting that WSB1 is specific to PD pathology.

## Discussion

Here we demonstrate that WSB1 is a novel interacting protein of LRRK2, and that WSB1 ubiquitinates LRRK2 through K27 and K29 ubiquitin linkages. Expression of WSB1 decreases LRRK2 soluble protein levels, and increases LRRK2 aggregation, and strikingly ameliorates mutant LRRK2 neuronal toxicity. Knocking down endogenous WSB1 in primary neurons exacerbates mutant LRRK2 neuronal toxicity, indicating a role for endogenous WSB1 in modulating LRRK2 cell toxicity. Furthermore, expression of WSB1 in a *Drosophila* G2019S overexpression model leads to LRRK2 protein aggregation, and ameliorates the dopamine neuron loss and motor phenotype. Knocking down WSB1 in *Drosophila* model of LRRK2 increases neuronal toxicity and decreases protein aggregation. Finally, WSB1 is found in Lewy bodies in human PD post-mortem tissue. These data indicate a role for WSB1 in modulation of mutant LRRK2 pathogenesis, and an involvement in Lewy body pathology in sporadic PD.

Cellular and *in vitro* ubiquitination assays and mutagenesis studies show that WSB1 robustly ubiquitinates WT and G2019S LRRK2 through K27 and K29 and not through K48. We show that WSB1 decreases soluble expression of LRRK2; however, it does not appear to lead to degradation through the proteasome, since the proteasome inhibitor MG132 did not return the levels of LRRK2 to baseline. We then demonstrate through biochemical fractionation and immunocytochemistry that WSB1 causes LRRK2 to become insoluble and aggregate.

To determine the functional consequences of these observations, we performed cell toxicity assays in primary neurons and show that WSB1 rescues G2019S LRRK2-induced toxicity. In addition, knocking down WSB1 leads to an increase in LRRK2 toxicity. We also show using both gain of function and loss of function approaches in a *Drosophila* model of LRRK2 that WSB1 modulates LRRK2 aggregation and toxicity.

Finally, we show that WSB1 is identified in almost all of the Lewy bodies observed in human sporadic PD post-mortem tissue. This suggests a role for WSB1 in sporadic PD, as well as LRRK2 PD. We suggest that WSB1 may be involved in the aggregation pathway in sporadic PD. While we identified LRRK2 in 25% of the Lewy bodies, there are reports of LRRK2 PD without alpha-synuclein-positive Lewy bodies or Lewy neurites, and it is unclear whether LRRK2 is a major component of Lewy body pathology^40^.

These data may suggest a broader role for ubiquitination in the pathogenesis of PD and other neurodegenerative diseases. DJ1 and huntingtin have been shown to aggregate through K27 and K29 ubiquitin linkages, but the functional consequences were not explored^41^,^42^. WSB1 rescues mutant LRRK2-induced neuronal death, and the mutant LRRK2 *Drosophila* phenotype. This is consistent with the idea that Lewy bodies and huntingtin inclusions^43^ are protective. Since aggregation is a core feature of neurodegenerative disorders, this has implications for mechanisms of other neurodegenerative diseases.

The data in this manuscript also suggest a biological function for the ubiquitin lysines linkages through K27 and K29. Since ubiquitination can occur through seven different lysines, it is likely that the different linkages lead to different cellular consequences. Here we demonstrate that K27 and K29 can signal proteins for aggregation. Ubiquitin has been known as a marker for inclusions in neurodegenerative disorders. We propose that K27 and K29 ubiquitin linkages constitute a signal leading to protein aggregation as a mechanism for the cell to protect itself from toxic proteins. Therefore, ubiquitin is a marker for inclusions because it is the signal for aggregation, and not just a byproduct of the aggregation pathway. It could be that these linkages are formed when the cell can no longer degrade a protein via K48 ubiquitination and the proteasome and, therefore, the neuron needs another way to protect itself. Alternatively, there may be a cellular signal for an abnormal protein to be ubiquitinated and aggregated through the atypical lysine chains versus ubiquitination through K48 and targeting to the proteasome. The nature of this signal, which could be alternative protein conformation, is a topic for future study.

Our data suggest an important neuronal protective role for WSB1 and K27 and K29 linkages. Furthermore, enzyme activities of E3 ligases like WSB1 are regulated, and these activities can be modulated^44^,^45^. Small molecules can serve to modulate enzyme activities. We propose WSB1 as a therapeutic target for LRRK2 PD and potentially for sporadic PD.

## Methods

### Yeast two-hybrid analysis

We used commercial systems (ProQuest system from Invitrogen) as well as vectors and libraries devised according to Chevray and Nathans^46^,^47^. For the yeast two-hybrid screen, we used a construct containing the kinase domain of LRRK2 with a point mutation to inactivate kinase activity^48^ cloned into the GAL4-binding domain vector (pDBleu). We screened a human substantia nigra library in pPC86 vector from Paul Worley's lab fused to the GAL4 activation domain. The yeast strain MAV 203 in the Invitrogen system was used. Experiments were conducted according to the manufacturer's instructions (Invitrogen). Positive clones were isolated by three reporter gene phenotypes (HIS3, URA3 and LacZ).

### Cells and transfections

HEK293-FT, N2a and NIH 3T3 cells (ATCC) were maintained using standard protocol. Twenty-four hours before tranfection, cells were seeded to obtain a final confluency of 70–90%. All transfections were carried out with Lipofectamine 2000 (Invitrogen) using standard manufacturer's protocols. LacZ or pcDNA was used as an expression control for WT, G2019S and WSB1 so equal amounts of DNA were transfected between those groups and LRRK2 plus WSB1.

### Co-immunoprecipitation

Co-transfected cells, non-transfected cells or mouse brain tissues were homogenized in PBS and 1% triton, supplemented with protease inhibitors cocktail (Complete). Protein G Sepharose beads (GE Healthcare) were used to preclear the lysate for 1 h and then the lysate was removed and incubated with an antibody to LRRK2 or WSB1 or the corresponding tag for 1 h. The lysate and antibody mixture was then incubated with protein G Sepharose overnight at 4 degrees. The next day, the beads were washed with lysis buffer three times, followed by two PBS washes, eluted with Laemmli buffer and heated at 95 degrees for 5 min. 20–50 ug of protein was resolved by SDS–PAGE and western blotting was performed.

### Antibodies

Anti-Flag and anti-Myc antibodies were from Sigma. Anti-LRRK2 antibody was from Epitomics (MJFF2) or NeuroMab (N138/6). Anti-WSB1 was from Santa Cruz (SC 393200) or Rabbit anti-WSB1 polyclonal antibodies were developed using a specific epitope to either amino acids 1–15 or amino acids 150–166 of WSB1, and affinity purified (Covance). Anti-alpha-synuclein was from BD Transduction Laboratories and anti-ubiquitin antibody was from Dako Cytomation. Antibodies were used at 1:1,000 dilution for western Blots.

### Ubquitination assays

We conducted a cellular ubiquitination assay using Flag-tagged LRRK2 and Myc-tagged WSB1, co-transfected in the presence of HA-tagged ubiquitin. Cells were lysed in 2% SDS buffer, boiled for 10 min and then sonicated. The lysate was diluted 1:10 with 1% triton buffer and centrifuged at 14,000 r.p.m. for 10 min. Lysate was immunoprecipitated for LRRK2 using the anti-Flag antibody as described above, and subjected to SDS–PAGE and western blotting using the Flag tag for LRRK2, Myc tag for WSB1 and HA tag for ubiquitin. We also performed an *in vitro* ubiquitination assay by immunoprecipitating LRRK2 (transfected in SH-SY5Y). LRRK2 was then incubated with 40 mM Tris (pH 7.6), 5 mM MgCl2, 2 mM dithiothreitol, 1 mM ATP, 10 mM phosphocreatine, 0.1 mg ml^−1^ creatine phosphokinase, 7.5 mg ubiquitin, 1 mM ubiquitin aldehyde, 100 ng of ubiquitin-activating enzyme and 200 ng of UbcH5b, in the presence or absence of 1 mg of recombinant WSB1. We incubated for 1 h at 37 degrees, washed four times with PBS and performed SDS–PAGE and western blotting with anti-ubiquitin and anti-Flag as control for the immunoprecipitation.

### Cold Sarkosyl insoluble pellet fraction

N2a cells were grown to 70% confluency in 100 mm plates and transfected with LRKK2, LRRK2 plus WSB1 or LRRK2 plus CHIP. After 48 h, cells were lysed with 900 μl of lysis buffer (50 mM HEPES, pH 7.5, 300 mM NaCl, 250 mM Sucrose, 5 mM EDTA, 5 mM glutathione (GSH), 1% NP-40, 0.2% Sarkosyl, 2 × PIs (Roche), 1 mM PMSF) and centrifuged for 30 min at 1,800*g*. The pellet was resuspended in 900 μl of high-salt buffer (50 mM HEPES, pH 7.5, 1.5 M NaCl, 250 mM Sucrose, 5 mM EDTA, 5 mM GSH, 1% NP-40, 0.2% Sarkosyl, 1 mM PMSF) and centrifuged for 30 min at 1,800*g*. The resulting pellet was resuspended and subjected to a DNA digest for 30 min at 37 °C in 50 mM Tris, pH 8, 250 mM Sucrose, 5 mM MgCl2, 5 mM GSH, 1% NP-40, 2 × PI (Roche), 1 mM PMSF and DNase I 40 U ml^−1^ and subsequently incubated overnight. Samples were then centrifuged for 30 min at 1,800*g* and resuspended in 900 μl of 50 mM HEPES and 0.2% Sarkosyl. Finally, samples underwent ultracentrifugation at 100,000*g* for 45 min at 4 °C (70 Ti rotor in Optima; Beckman Coulter), and the resulting pellets were solubilized in loading buffer. All steps were performed on ice unless otherwise stated.

### Immunofluorescence

Mouse Neuro-2a (N2a) cells (ATCC)were plated on coverslips, cultured overnight and transfected as described above. Cells were fixed with 4% paraformaldehyde and then permeabilized with 0.25% triton for 5 min. Cells were washed three times in PBS and blocked for 1 h in 5% normal goat serum. Cells were incubated with antibodies to LRRK2 or WSB1 or their corresponding tags overnight at 4 degrees (all antibodies were used at a 1:250 dilution, except for the flag antibody that was used at a 1:500 dilution). Washed cells were incubated with fluorescent secondary antibody (Invitrogen) for 1 h. Cover slips were mounted with Vectashield containing DAPI (Vector Laboratories). Cells were viewed on a Zeiss confocal microscope.

### Cell viability studies in primary cortical neurons

Primary cortical neurons isolated from embryonic day 16 mice were at DIV6. After 48 h, cells were fixed with 4% paraformaldehyde. After three washes with PBS, cells were treated with 0.8 μg ml^−1^ of bisbenzimide (Hoechst 33342, Sigma). Cells were automatically analysed using an inverted fluorescence microscope (Axiovert 200, Zeiss) and images were digitized from 144 independent fields per well. Transfected cells were visualized by immunostaining with anti-Flag antibody and quantified for cell survival using the Volocity software (Perkin Elmer) by automated measurement of the average intensity of Hoechst-stained nuclei of transfected cells. Cells were considered as viable when their intensity was lower than 200% of the control intensity. All animal studies were performed in compliance with ethical regulations as approved by the Johns Hopkins University Animal Care and Use Committee.

### WSB1 knockdown in primary cortical neurons shRNA efficacy

Primary cortical neurons isolated from embryonic day 16 mice were at DIV 6. After 24 h, cells were fixed and treated similarly to the cell viability studies. To determine the efficacy of the shRNA to WSB1, cells were identified by green fluorescent protein (GFP)-positive staining and the WSB1 staining was measured in the soma of the cells using an antibody to WSB1. For comparison, a GFP-negative cell was measured within the same field using Hoechst staining for identification. Each transfected cell measurement was then normalized to their non-transfected paired control.

### Cell viability studies in primary neurons using shRNA

Primary cortical neurons isolated from embryonic day 16 mice were transfected at DIV 6. After 24 h, cells were fixed and treated and analysed similarly to the cell viability studies described above.

### *Drosophila*-based studies

Fly lines for *elav*-Gal4 (pan-neuronal) and *ddc*-Gal4 (dopaminergic neuron-specific) were purchased from Bloomington Drosophila Stock Center (Bloomington, IN, USA). UAS-LRRK2-G2019S transgenic flies were previously described^49^. Immunohistochemical analysis of whole-mount adult fly brains were prepared according to published protocols^49^ and stained with rabbit anti-TH (1:300, Pel-Freez Biologicals, Milwaukee), anti-WSB1 or anti-elav (1: 10, Developmental Studies Hybridoma Bank) as primary antibodies. The stained samples were viewed using an Olympus Fluoview Upright Confocal Microscope. DA neurons were quantified according to published method^50^. Climbing assays were carried out as follows: 20 female adult flies from each group were randomly selected after anaesthetisation and placed in a vertical plastic column (length 25 cm; diameter 1.5 cm). Age-matched normal flies were used as controls. After a 2-h recovery period from CO_2_ exposure, flies were gently tapped to the bottom of the column and the number of flies that reached the top of column at 1 min was counted. Results are presented as mean+s.e.m. of the scores obtained from three independent experiments.

RNAi-WSB1 flies were purchased from Venna *Drosophila* RNAi Centre (VDRC) stock #12588 (WSB-1 RNAi #1) and stock #12589 (WSB-1 RNAi #2) and crossed with G2019S before staining with anti-LRRK2 antibodies (Novus, 1:100) and anti-elav (DSHB, 1:10) at age 60 days. We observed in line #1, an ∼40% loss of WSB-1 expression. This line was used for all the subsequent studies. Reverse transcription–PCR Primers for WSB-1 and GAPDH: WSB-1 Forward primer- 5′-CCGGTTTGCTTTGTTGTTTT-3′, WSB-1 Reverse primer- 5′-GCACTTGAACAGCCTTGACA-3′, GAPDH Forward primer- 5′-ATCGTCGAGGGTCTGATGAC-3′, GAPDH Reverse primer- 5′-CGGACGGTAAGATCCACAAC-3′.

### Immunohistochemistry

Tissue from at least three PD cases and from substania nigra were used for immunohistochemistry studies. Tissues sections were deparaffinized, treated with formic acid for 5 min and then H_2_O_2_. Slides were microwaved and cooled slowly with water and then blocked with 3% normal goat serum. Tissue was then incubated with primary antibody overnight at 4 degrees. Tissue was washed and incubated with fluorescent secondary antibody for 1 h. Tissue was then mounted with Vectashield containing DAPI (Vector Laboratories).

Blots have been cropped for presentation purposes. Full-length versions are available in Supplementary Figs 3 and 4.

The authors declare that the data supporting the findings of this study are available within the article and its supplementary information files.

## Additional information

**How to cite this article:** Nucifora, F. C. *et al*. Ubiqutination via K27 and K29 chains signals aggregation and neuronal protection of LRRK2 by WSB1. *Nat. Commun.* 7:11792 doi: 10.1038/ncomms11792 (2016).

## Supplementary Material

Supplementary Information Supplementary Figures 1-4
