# Supplementary material for: Ubiqutination via K27 and K29 chains signals aggregation and neuronal protection of LRRK2 by WSB1
Source: Nat Commun. 2016 Jun 7;7:11792. doi: 10.1038/ncomms11792 (PMC4899630; doi:10.1038/ncomms11792)
Supplement: Supplementary Information — Supplementary Figures 1-4 [file ncomms11792-s1.pdf]

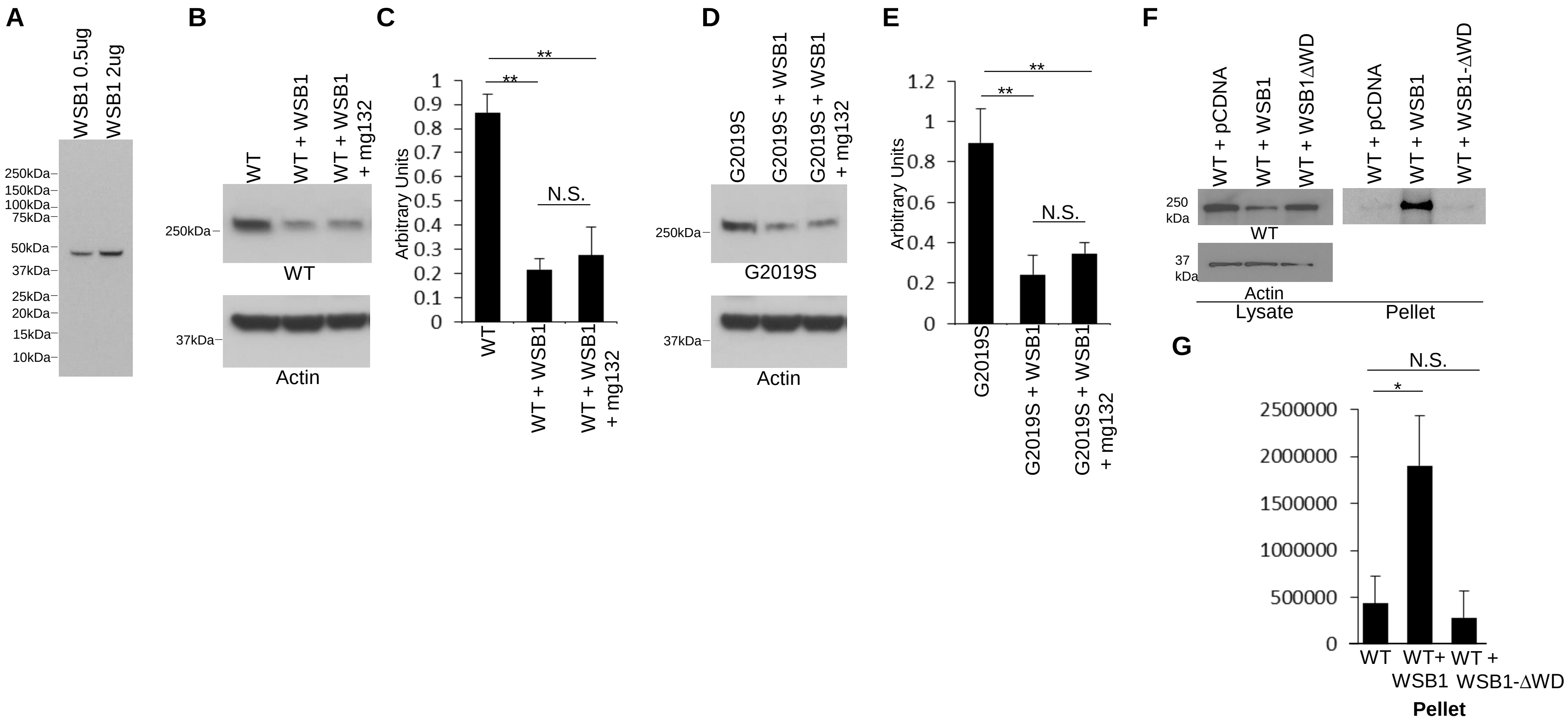

Supplementary Figure 1. A) Validation of the WSB1 antibody. Antibody recognizes a single band at the appropriate predicted molecular weight for WSB1. B and D) HEK 293 cells were transfected with LRRK2 alone or with WSB1 with and without the proteasome inhibitor MG-132. Cell lysates were run on an SDS-PAGE gel and Western blotted for LRRK2. C and E) Quantification of B and D respectively. F) Sarkosyl detergent fractionation in N2a cells transfected with WSB1- ΔWD. G) Quantification of experiments from E. WSB1- ΔWD has no effect on LRRK2 insoluble levels. All experiments were performed on at least three independent experiments where data are the mean +/- SD. *P<0.05, ** P<0.001 using a one-way ANOVA test.

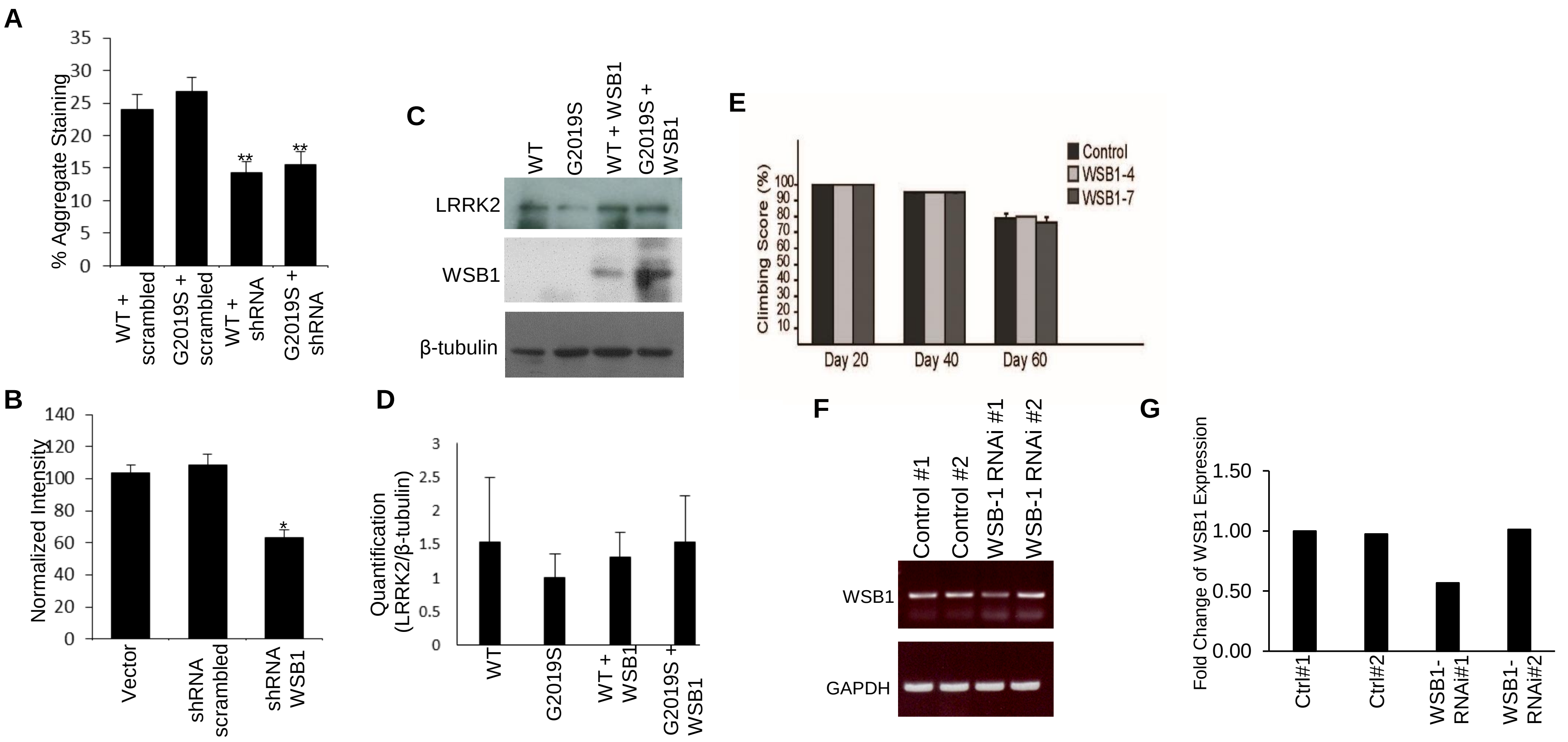

Supplementary Figure 2. A) N2a cells were transfected with LRRK2 and shRNA to WSB1 or scrambled shRNA and percentage of cells with aggregates were quantified. B) Quantification of WSB1 shRNA efficacy on WSB1 protein expression compared to WSB1 expression with scrambled shRNA or vector control in primary neurons. C) Western blot of total homogenate protein for the different drosophila models. D) Quantification of H. This indicates that there was not a significant difference in expression between drosophila models. E) Analysis of climbing scores for WSB1 flies compared to control. F and G) RT-PCR results of knockdown of WSB-1 in the fly brain using elav-GAl4 driver. F) Gel showing a decrease in expression of WSB1-RNAi#1 but not WSB1-RNAi#2 compared to control. G) Quantification of M. All experiments were performed on at least three independent experiments where data are the mean +/- SD. *P<0.05, ** P<0.001 using a one-way ANOVA test.

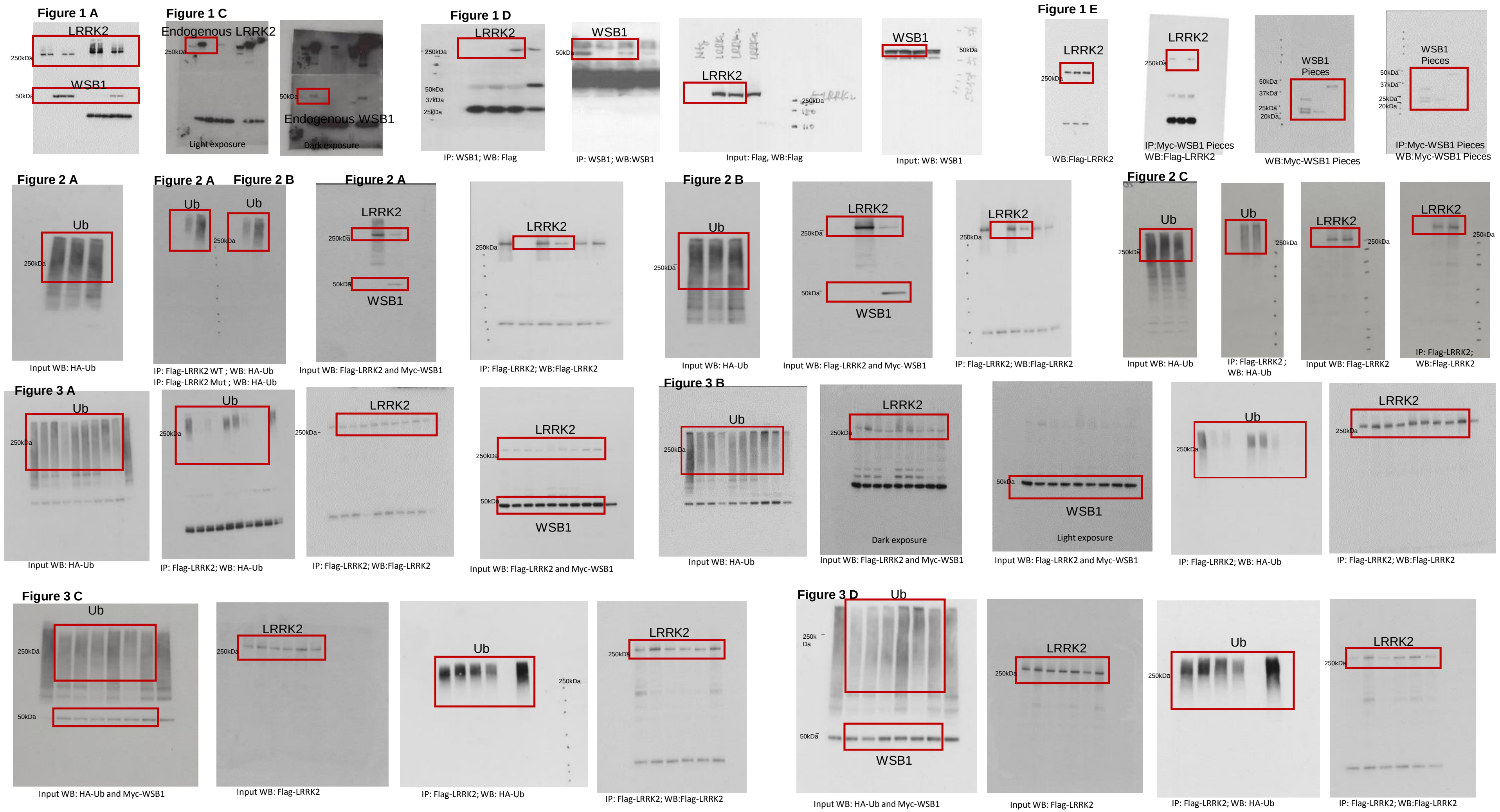

Supplementary Figure 3. Full version of blots

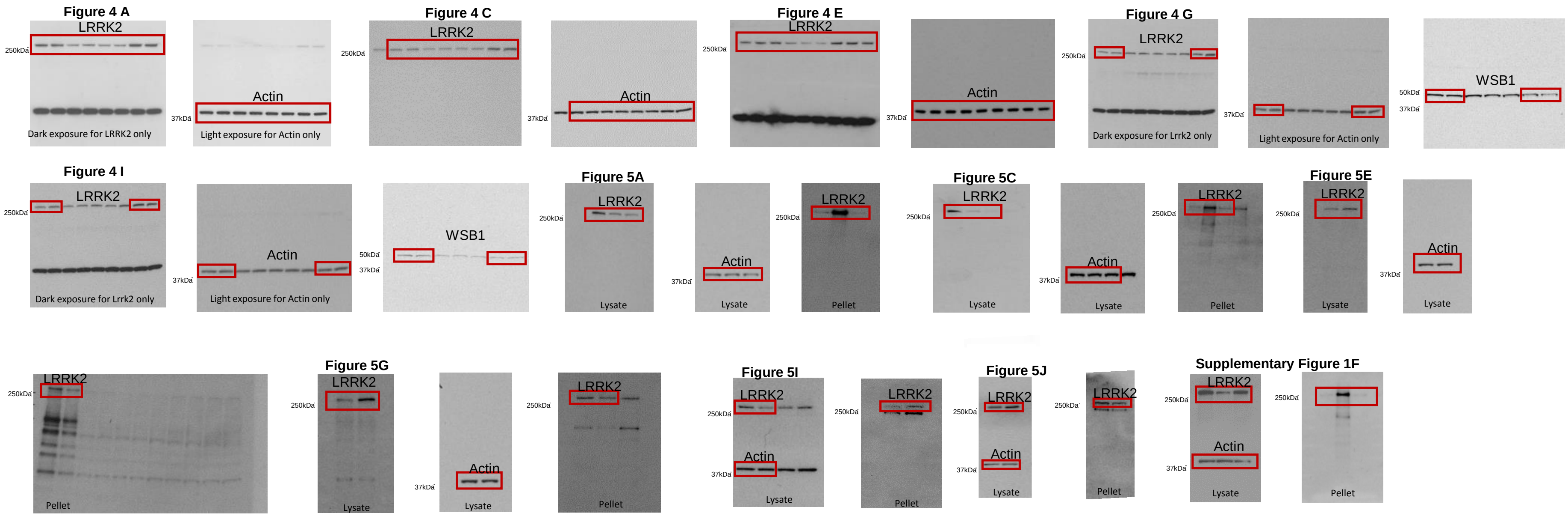

Supplementary Figure 4. Full version of blots
